# Apatinib potentiates irradiation effect via suppressing PI3K/AKT signaling pathway in hepatocellular carcinoma

**DOI:** 10.1186/s13046-019-1419-1

**Published:** 2019-11-06

**Authors:** Junbin Liao, Huilin Jin, Shaoqiang Li, Lixia Xu, Zhenwei Peng, Guangyan Wei, Jianting Long, Yu Guo, Ming Kuang, Qi Zhou, Sui Peng

**Affiliations:** 1 Department of Liver Surgery, The First Affiliated Hospital, Sun Yat-sen University, Guangzhou, 510080 China; 2 Guangdong Research Institute of Gastroenterology, The Sixth Affiliated Hospital, Sun Yat-Sen University, Guangzhou, 510080 China; 3 Department of Oncology, The First Affiliated Hospital, Sun Yat-sen University, Guangzhou, 510080 China; 4 Precision Medicine Institute, The First Affiliated Hospital, Sun Yat-sen University, Guangzhou, 510080 China; 5 Department of Radiation Oncology, The First Affiliated Hospital, Sun Yat-sen University, Guangzhou, 510080 China; 6 Clinical Trials Unit, The First Affiliated Hospital, Sun Yat-sen University, Guangzhou, 510080 China; 7 Department of General Surgery, The First Affiliated Hospital, Sun Yat-sen University, Guangzhou, 510080 China; 8 Division of Interventional Ultrasound, The First Affiliated Hospital, Sun Yat-sen University, Guangzhou, 510080 China; 9 Department of General Surgery, Huiya Hospital of The First Affiliated Hospital, Sun Yat-sen University, Huizhou, 516081 Guangdong China; 10 Department of Gastroenterology and Hepatology, The First Affiliated Hospital, Sun Yat-sen University, Guangzhou, 510080 China

**Keywords:** Hepatocellular carcinoma, Apatinib, Irradiation, Radiosensitization, PI3K/AKT pathway

## Abstract

**Background:**

Limited effective intervention for advanced hepatocellular carcinoma (HCC) is available. This study aimed to investigate the potential clinical utility of apatinib, a highly selective inhibitor of the vascular endothelial growth factor receptor-2 (VEGFR2) tyrosine kinase, as a radiosensitizer in the treatment of HCC.

**Methods:**

Four human HCC cell lines SMMC-7721, MHCC-97H, HCCLM3 and Hep-3B were treated with apatinib, irradiation or combination treatment. Colony formation assay, flow cytometry and nuclear γ-H2AX foci immunofluorescence staining were performed to evaluate the efficacy of combination treatment. RNA sequencing was conducted to explore the potential mechanism. The impact of combination treatment on tumor growth was assessed by xenograft mice models.

**Results:**

Colony formation assay revealed that apatinib enhanced the radiosensitivity of HCC cell lines. Apatinib suppressed repair of radiation-induced DNA double-strand breaks. Flow cytometry analysis showed that apatinib increased radiation-induced apoptosis. Apatinib radiosensitized HCC via suppression of radiation-induced PI3K/AKT pathway. Moreover, an in vivo study indicated apatinib combined with irradiation significantly decreased xenograft tumor growth.

**Conclusions:**

Our results indicate that apatinib has therapeutic potential as a radiosensitizer in HCC, and PI3K/AKT signaling pathway plays a critical role in mediating radiosensitization of apatinib.

## Background

Hepatocellular carcinoma (HCC) is one of the most prevalent malignancies and the third leading cause of cancer-related deaths worldwide [1]. Surgical resection, liver transplantation, and radiofrequency ablation (RFA) have been successfully developed as radical therapeutics for early stage HCC patients with well-preserved liver function [2]. However, those curative treatments are not always applicable, limited by the extent of disease, patient co-morbidities, tumor location and source of organs and so on. Moreover, over half of HCC is diagnosed at an advanced stage [3], with few effective treatments available.

Stereotactic body radiotherapy (SBRT), a non-invasive treatment, has been introduced into the treatment of HCC, even for advanced cases, and its efficacy and safety have been well characterized by several studies. SBRT provides excellent local control for HCC smaller than 2 cm, and overall survival at 1 and 2 years after SBRT was 74.1 and 46.3%, respectively [4]. More importantly, SBRT is still an effective treatment for patients with locally advanced primary liver malignancies. Recently, Bujold, et al. [5] reported that after SBRT treatment, the 1-year survival rate and local control rate of 102 patients with advanced HCC reached 55 and 87%, respectively, with a low risk of serious toxicity. With advances in radiation oncology technology, radiotherapy (RT) has now been recommended by the National Comprehensive Cancer Network Guidelines as a locoregional treatment option for inoperable HCC. Though intra- and extrahepatic spreading are still the predominant failure patterns for SBRT [4], it is worthwhile to explore how to increase its treatment potency, considering the abovementioned advantages of SBRT. SBRT in combination with other local or systemic therapies has been considered to be effective tactics to augment therapeutic potency [6, 7].

Sorafenib, a multitargeted tyrosine kinase inhibitor (TKI) and the only FDA-approved first-line targeted drug for patients with advanced HCC currently, yields to extend overall survival by less than 3 months in 2 large phase III clinical trials [8, 9]. More importantly, increasing clinical evidences indicate that radiotherapy and tyrosine kinase inhibitor therapy have synergistic antitumor effect. Yoshiyuki et al. [10] showed radiotherapy in combination with sorafenib dramatically prolonged overall survival of patients with advanced HCC compared to sorafenib alone (31.2 months vs. 12.2 months). However, SBRT combined with sorafenib has a high incidence of severe toxic side effects [11], which is intolerable for patients. However, apatinib [12], a novel tyrosine kinase inhibitor, highly selectively inhibits vascular endothelial growth factor receptor-2 tyrosine kinase (VEGFR-2) activity and thereby suppresses tumor growth by inhibiting tumor angiogenesis. Some studies [13–16] have revealed that apatinib shows encouraging antitumor activities and tolerable toxicities in several solid tumors, including HCC. Although well tolerated in patients with advanced HCC, apatinib monotherapy has limited control over HCC [17, 18], necessitating combinatorial administration of apatinib with other therapies for efficacy augmentation. Apatinib combined with chemotherapy or immunotherapy has achieved promising efficacy in several tumors [14, 15, 19, 20]. Several clinical studies [21, 22] have indicated favorable efficacy of the combined treatment of apatinib with radiotherapy with controllable and tolerable adverse reactions. However, whether radiotherapy combined with apatinib can bring clinical benefits to patients with HCC and its underlying mechanisms remain unknown.

Therefore, our study aimed to explore the effect of apatinib combined with radiotherapy on HCC through both in vitro and in vivo assays, investigate the potential mechanism, and provide a strong theoretical basis for exploration of clinical combinations of radiation and apatinib in HCC. In present study, we showed that apatinib improved the radiosensitivity of HCC cells by inhibiting PI3K/AKT pathway.

## Materials and methods

### Cell culture and Apatinib preparation

Human hepatocellular carcinoma cell lines SMMC-7721, MHCC-97H, HCCLM3, Hep-3B were purchased from the Cell Bank of the Chinese Academy of Sciences (Shanghai, China) and confirmed by STR. Cells were either maintained in RPMI 1640 or Dulbecco’s Modified Eagle’s Media (DMEM, Sigma, USA) supplemented with 10% fetal bovine serum (FBS, Gibco, NY, USA) and 1% penicillin–streptomycin.

Apatinib was obtained from Jiangsu Hengrui Medicine Co., Ltd. (Nanjing, China). Apatinib was dissolved in dimethyl sulfoxide (DMSO) for in vitro studies or in 0.5% (w/v) carboxymethyl cellulose (CMC) for in vivo experiment.

### Cell irradiation

HCC cells pretreated with/without apatinib for 24 h at 37 °C were exposed to ionizing radiation using X-ray linear accelerator (RS2000, RadSource, Suwanee, USA) at a dose rate of 1.24 Gy/min.

### Cell viability assay

Four HCC cells were seeded into 96-well plates at 4 × 10^3^ cells/well, respectively. After 24 h, cells were exposed to apatinib at various concentrations in fresh complete medium for another 48 h. The cell viability was determined using the Cell Counting Kit-8 (CCK-8, Dojindo, Japan) according to the manufacturer’s protocol. Each group was set up in quadruplicate.

### Colony formation assay

HCC cells were seeded into six-well plates and allowed to attach for 24 h. After 24 h pretreatment with DMSO or apatinib, supernatant was changed with fresh medium and cells were irradiated with different doses of X-ray radiation (2–8 Gy). Two to three weeks after irradiation, cells were fixed with 4% paraformaldehyde for 20 min followed by staining with 0.5% crystal violet at room temperature for 10 min. The colonies containing ≥50 cells per dish were counted. Cell survival curves were fitted with the multi-target, single-hit model. Sensitizing enhancement ratio (SER) was determined as the ratio of the mean inactivation dose under radiation-only conditions divided by the mean inactivation dose after apatinib plus irradiation treatment.

### Cell cycle and apoptosis analysis

For cell cycle analysis, 12 h after irradiation, cells were collected and then fixed with 70% ice-cold ethanol at − 20 °C overnight. Before flow cytometric analysis, cells were washed with PBS and then cocultured with 0.25 mg/ml RNase A and 50 μg/ml propidium iodide (PI) for 30 min at 37 °C in the dark. Samples were analyzed by flow cytometry (CytoFlex, BD Biosciences, USA) and data were analysed using ModFit 5.1 software. For apoptosis analysis, cells were collected, washed with PBS, and stained with Annexin V-FITC and PI (Annexin V, FITC Apoptosis Detection Kit, Dojindo, Japan) for 15 min at 37 °C in the dark. Samples were analyzed by flow cytometry and data were analysed using FlowJo 10.0 software. The Annexin-V+/PI- cells were in the early phase of the apoptotic process; the Annexin-V+/PI+ cells indicated late apoptosis. The percentages of both groups of cells were computed. Each group was set up in triplicate.

### Immunofluorescence

Approximately, 4 × 10^4^ cells were seeded on coverslips in 24-well plates and pretreated with apatinib or DMSO for 24 h and then irradiated with 4 Gy X-ray. After fixed in 4% paraformaldehyde, cells were blocked with 5% goat serum, then incubated with anti-Phospho-Histone H2AX antibody overnight at 4 °C and Alexa Fluor488-conjugated anti-rabbit antibody (Invitrogen, USA) for detection. After stained with DAPI (Fluoroshied with DAPI, Sigma, USA), samples were photographed with immunofluorescence microscopy.

### Western blots

Cell extracts were prepared on ice using cell lysis buffer (KeyGEN BioTECH, Nanjing, China) and phosphatase inhibitors (Sigma) as needed, boiled for 10 min under reducing conditions, and frozen at − 20 °C until use. 10–40 μg of protein were electrophoresed in 8% or 12% sodium dodecyl sulfate–polyacrylamide gel electrophoresis (SDS-PAGE) and blotted onto Immobilon PVDF membranes (Millipore). Signals were visualized by western blotting using primary antibodies (Additional file 1: Table S1), followed by corresponding peroxidase-conjugated secondary antibodies and Immobilion Western Chemilum HRP Substrate (Millipore, USA).

### RNA-sequencing

RNA from SMMC-7721 and MHCC-97H cells treated with 4 Gy radiation alone or radiaton plus apatinib was isolated and enriched for mRNA with MicroPoly (A) Purist (Ambion, Austin, TX, USA). The quality and quantity of mRNA were evaluated with a Bioanalyzer 2100 (Agilent Technologies, Santa Clara, CA, USA), and 0.5–1 mg mRNA for each sample was used for RNA sequencing (Illumina HiSeq 2000 platform). The significance of digital gene expression profiles was used to identify differential expression genes between the two groups. The pathway enrichment analysis was performed in Kyoto Encyclopedia of Genes and Genomes (KEGG) pathways using KOBAS software.

### Immunohistochemistry and TUNEL assay

For IHC, deparaffinized tumor sections were treated with sodium citrate buffer (pH 6.0) for antigen retrieval at boiling temperature for 2.5 min, and then 3% hydrogen peroxide for 10 min to quenched endogenous peroxidase activity, blocked and incubated with primary antibodies at 4 °C overnight. The Vectastain Elite ABC Kit (Vector Laboratories, Inc. USA) and DAB kit (Dako, Denmark) were used to amplify and detect signals. In Situ Cell Death Detection Kit, Fluorescein (Roche, USA) was used for TUNEL assay to examine the apoptosis rate in the tumor tissues. The deparaffinized sections were pretreated with Proteinase K for 30 min, quenched endogenous fluorescent signal, and then incubated in TUNEL reaction mixture for 1 h at 37 °C. The nucleus was stained with DAPI. Apoptosis rate was calculated in 5 microscopic fields of each sample (four samples from each group).

### In vivo study

Male BALB/c nude mice (3–4 weeks of age) were housed in specific pathogen-free facilities. The protocols involving animals were approved by the Institutional Ethics Committee for Clinical Research and Animal Trials of the First Affiliated Hospital, Sun Yat-sen University. SMMC-7721 (5 × 10^6^) cells were inoculated into the right flanks of mice. After 7 days, mice were randomly divided into four groups (*n* = 8): (1) carboxymethyl cellulose (CMC) (negative control); (2) Apatinib; (3) CMC + irradiation; and (4) Apatinib + irradiation. CMC and apatinib were administered via oral gavage at doses of 150 mg/kg once a day. Tumors were irradiated with 7 Gy X-ray using RS2000 X-ray linear accelerator at a dose rate of 1.24 Gy/min on day 13 and 15. Tumor dimensions were measured every 3 days, and the tumor volume was calculated using V = length×width^2^/2. At day 34, mice were euthanized and tumor tissues were harvested for TUNEL and immunohistochemistry detection.

### Statistical analysis

All means were calculated from at least three independent experiments, with error bars representing the SEM. Two-way ANAVO analysis of variance and unpaired t-test were performed using GraphPad Prism 5.0 (GraphPad Software, San Diego, USA). Group differences were considered statistically significant when *p*<0.05.

## Results

### Apatinib enhances radiosensitivity of HCC cells

**Fig. 1:**
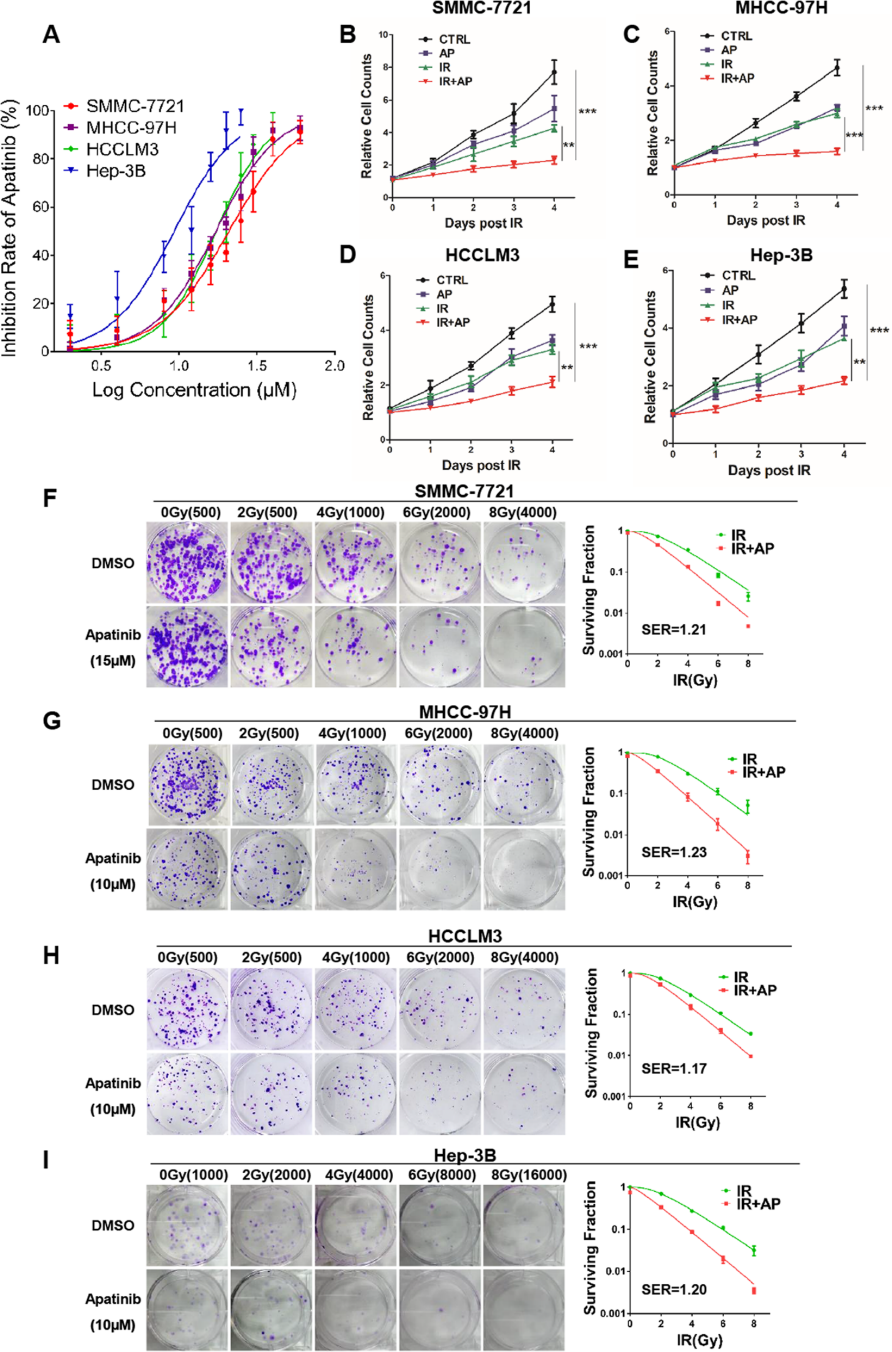
Apatinib enhances radiosensitivity of HCC cell lines in vitro. **a** After 48 h’s incubation with apatinib at various concentrations, cell viability of HCC cell lines SMMC-7721, MHCC-97H, HCCLM3 and Hep-3B was detected by CCK-8 kit, respectively. **b**-**e** After treatment with apatinib for 24 h, followed by 4 Gy radiation exposure, viable cells were counted using the trypan blue dye exclusion method. **f**-**i** After apatinib treatment, sensitization of irradiation (IR)-treated SMMC-7721, MHCC-97H, HCCLM3 and Hep-3B cells was evaluated through colony formation assay, respectively. The sensitivity enhancement ratio (SER) was measured using the multi-target, single-hit model. Data is presented as the mean ± SEM, *p < 0.05, ** *p* < 0.01

To determine the effect of apatinib on growth of HCC cell lines, SMMC-7721, MHCC-97H, HCCLM3, Hep-3B were incubated with different concentrations of apatinib for 48 h, and then cell viability was detected by Cell Counting Kit-8 (CCK-8) assays. We observed that the cell viability was suppressed by apatinib in a dose-dependent manner (Fig. 1a). The IC50 was 23.8 μM, 17.2 μM, 20.9 μM and 14.8 μM in SMMC-7721, MHCC-97H, HCCLM3 and Hep-3B, respectively. Based on the cell viability, 15 μM apatinib was used for SMMC-7721 and 10 μM apatinib was selected for MHCC-97H, HCC-LM3, and Hep-3B for further experiments.

The inhibitory effect of apatinib in combination with irradiation on the growth of HCC cells was assessed by cell counting assay. SMMC-7721, MHCC-97H, HCCLM3, Hep-3B cells were pretreated with apatinib or DMSO for 24 h and then exposed to 4 Gy or sham radiation. As compared to irradiation alone, cell growth was remarkably suppressed in apatinib pretreatment plus X-ray group at different time points, especially at day 4 (Fig. 1b-e). To further confirm the radiosensitizing effects of apatinib, colony formation assay was performed. Colony formation revealed pretreatment with apatinib plus 4 Gy X-ray irradiation significantly reduced the survival rate in all four HCC cell lines, as compared with cells treated with irradiation alone. The sensitivity enhancement ratio (SER) was 1.22, 1.22, 1.19 and 1.24 in SMMC-7721, MHCC-97H, HCCLM3 and Hep-3B, respectively (Fig. 1f-i). These data indicated that apatinib sensitized HCC cell lines to irradiation in vitro.

### Apatinib suppresses repair of radiation-induced DNA double-strand breaks

**Fig. 2:**
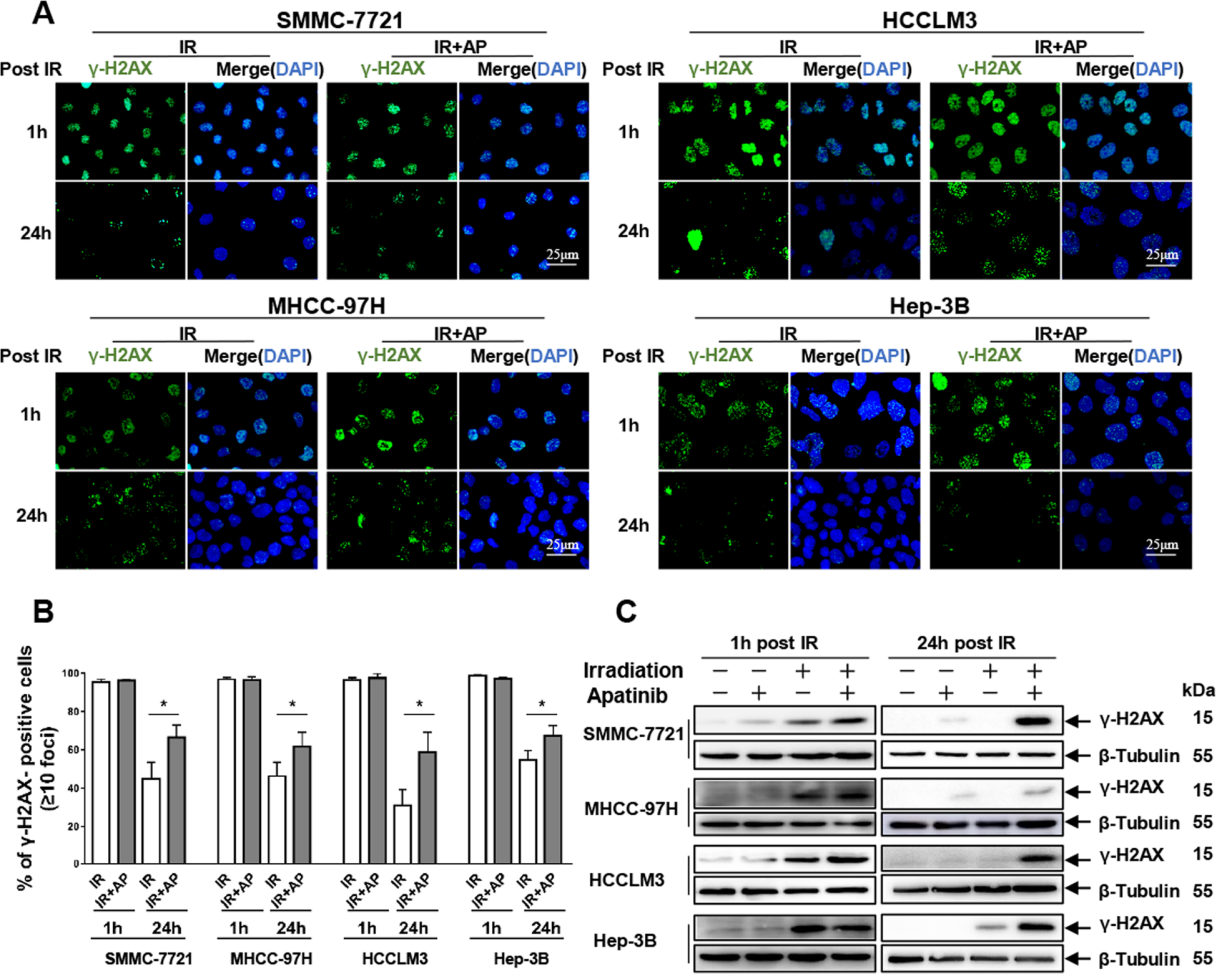
Apatinib suppresses repair of radiation-induced DNA double-strand breaks. **a** The representative images of γ-H2AX foci immunofluorescence staining in SMMC-7721, MHCC-97H, HCCLM3 and Hep-3B cells treated with IR (4 Gy) or in combination with apatinib at 1 h and 24 h after IR. **b** Cells with more than 10 γ-H2AX foci were counted and then quantified. **c** Western blot showed the combination treatment further enhanced γ-H2AX formation 1 h and 24 h after IR. Error bars indicate SEM, *p < 0.05

The major cellular impact of ionizing radiation is to induce DNA double-strand breaks (DSBs) and trigger the DNA damage repair response [23]. To determine whether apatinib pretreatment would enhance radiation-induced DNA damage and interfere with the DNA damage repair process, immunofluorescence was performed to evaluate foci of phosphorylated histone H2AX (γ-H2AX), which is a sensor of DNA strand breaks and promotes efficient DSBs repair [24]. We found that almost 100% HCC cells were γ-H2AX positive regardless of irradiation or combination treatment at an early time points (1 h post IR). In contrast, γ-H2AX positive cell remained notably more in apatinib pretreatment plus irradiation than that in irradiation alone at 24 h post IR (66.7% ± 3.8% compared to 45% ± 4.9% in SMMC-7721, and 61.7% ± 4.3% compared to 46.3% ± 4.1% in MHCC-97H, 58.7% ± 6.1% compared to 31% ± 4.7% in HCCLM3, 67.3% ± 2.7% compared to 54.7% ± 2.9% in Hep-3B, all *p*<0.05, Fig. 2a-b), which reflected inefficient repair of DSBs. In addition to immunofluorescence, we observed parallel increases in double-strand DNA breaks at 1 h and 24 h post IR as detected by Western blot (Fig. 2c). Together, these results suggested that apatinib reduced the DNA damage repair activity in HCC cell lines.

### Apatinib increases radiation-induced apoptosis in HCC cells

Radiation-induced DNA damage triggers cell cycle G1 or G2 arrest, allowing time for cells to repair DNA damage. Apatinib has also been reported to induce cell cycle arrest at G1 or G2 arrest [25, 26]. To investigate whether the radiosensitization of apatinib was related to cell cycle redistribution, PI staining using flow cytometry was performed to assess the DNA content. 4 Gy IR alone induced a typical G2/M-phase arrest in all four HCC cell lines after 24 h, while apatinib increased the G0/G1 population. For SMMC-7721 cell, the radiation-induced G2/M-phase arrest was further enhanced by combination with apatinib, while such effect didn’t exist in other three cell lines (Additional file 2: Figure S1).

**Fig. 3:**
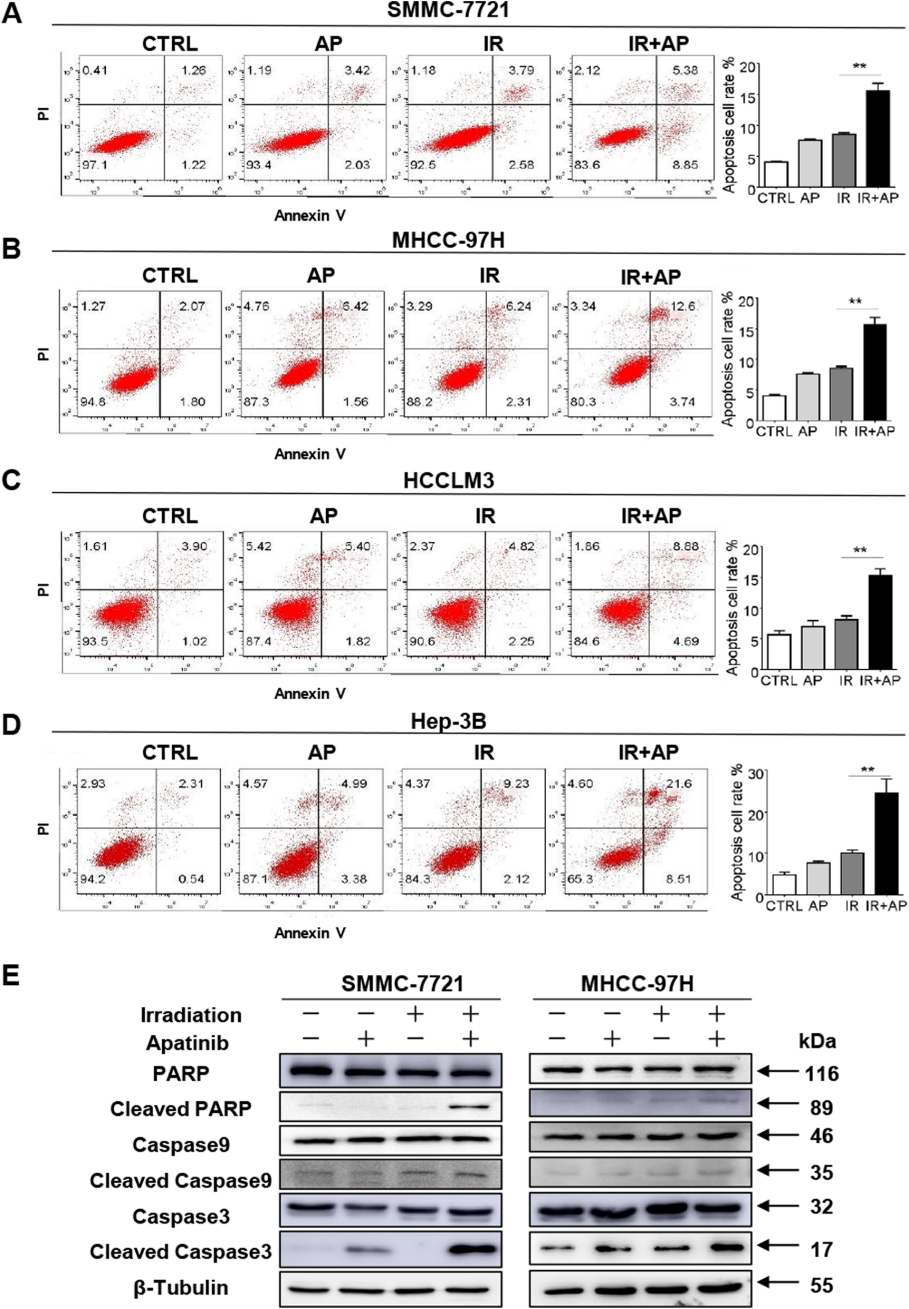
Apatinib increases radiation-induced apoptosis in HCC cells. **a**-**d** Apoptosis was measured at 48 h after IR by FACS using Annexin V FITC/PI double staining assay in HCC cells pretreated with apatinib. **e** Western blot analysis showed the effects of combination treatment of apatinib with irradiation on the apoptosis markers in SMMC-7721 and MHCC-97H cells. Data is presented as mean ± SEM, *p < 0.05, ** *p <* 0.01

To further investigate the reason behind the growth inhibition induced by radiosensitization of apatinib in HCC cell lines, flow cytometry and Western blot were used to determine the level of apoptosis. Cell apoptosis was assessed quantitatively by flow cytometry after Annexin V-FITC and PI staining. The results showed that 4 Gy X-rays alone moderately increased the apoptotic population of SMMC-7721, MHCC-97H, HCCLM3, Hep-3B cells compared with sham-treatment cancer cells. Pretreatment with apatinib remarkably enhanced the radiation-induced apoptosis in SMMC-7721, MHCC-97H, HCCLM3, Hep-3B cells, as compared to the radiation group (14.41% ± 1.097, 15.63 ± 1.21, 15.25 ± 1.08 and 24.55% ± 3.441%, vs 7.87% ± 0.75, 8.5% ± 0.29, 8.08 ± 0.61, 10.14% ± 0.67%, respectively, all *p*<0.01) (Fig. 3a-d). Consistently, apatinib pretreatment further increased the level of cleaved caspase 3 and cleaved caspase 9, cleaved-PARP, which are key indicators of apoptosis (Fig. 3e). Taken together, these results demonstrated that apatinib enhanced radiation-induced cell apoptosis.

### Apatinib blocks PI3K/AKT pathway to sensitize HCC cells to radiation

**Fig. 4:**
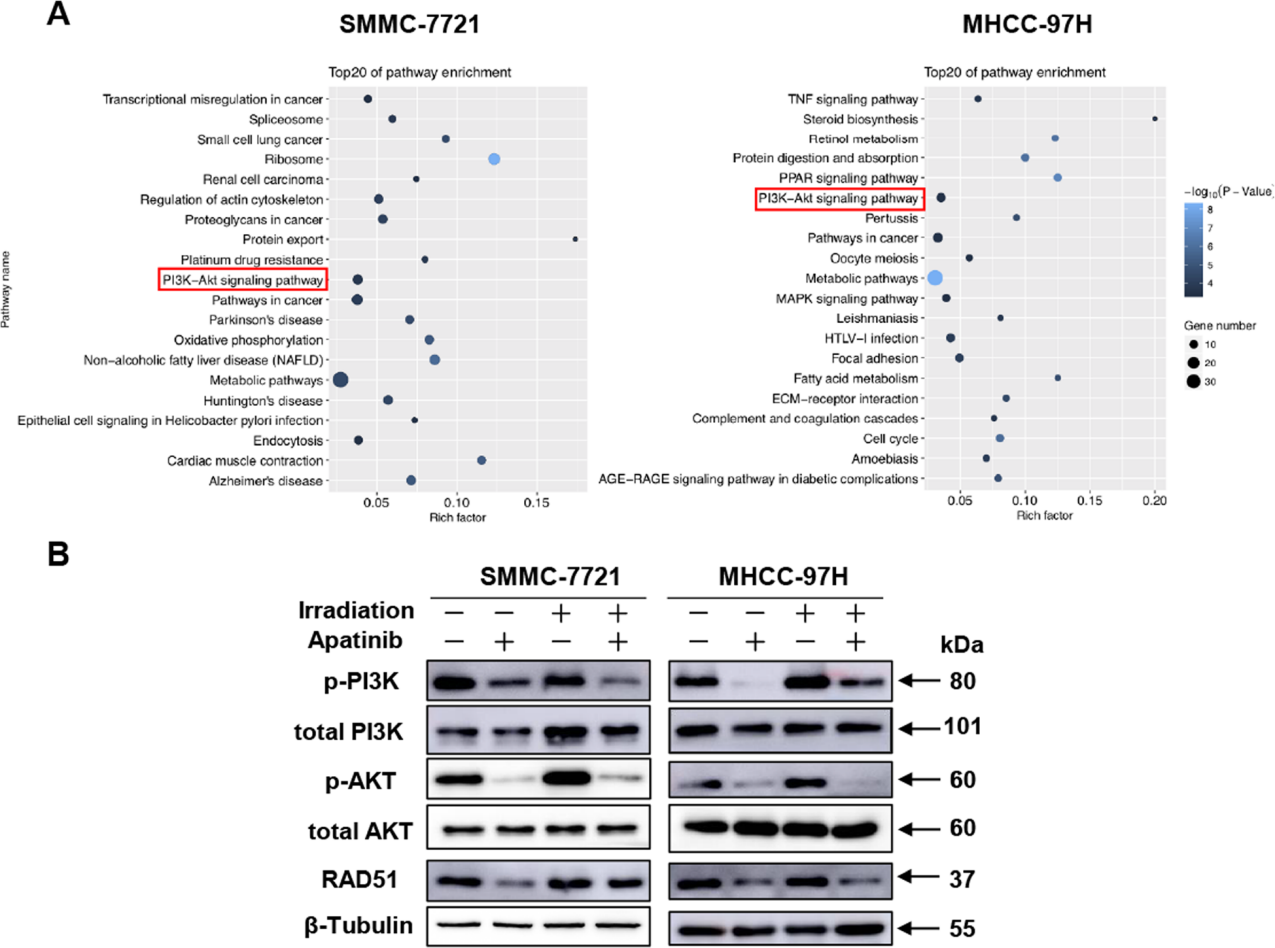
Apatinib blocks PI3K/AKT pathway activated by irradiation. **a** Pathway enrichment analysis of comparing the irradiation (4 Gy) group and the combined group through RNA-sequencing. **b** Protein expression of important genes in PI3K/AKT signaling by Western blot at 24 h after radiation. Data is presented as mean ± SEM, *p < 0.05

To explore the potential mechanism how apatinib regulates radiosensitivity, we performed RNA-sequencing in combined treatment group and radiation group using SMMC-7721 and MHCC-97H cells. As shown in Fig. 4a, pathway enrichment analysis revealed that PI3K-AKT signaling pathway was significantly affected in both SMMC-7721 and MHCC-97H cell lines. It is reported that PI3K/AKT cascade is up-regulated by radiation involved in mechanism of radiation resistance, and impaired PI3K/AKT signaling pathway can inhibit DNA double-strand break repair and improve radiosensitivity [27, 28]. We therefore speculated that apatinib might affect the radiosensitivity of HCC cell lines through inhibition of PI3K/AKT signaling pathway. Western blot analysis revealed that apatinib decreased the level of phosphorylated PI3K and phosphorylated AKT in both SMMC-7721 and MHCC-97H cell lines. Although PI3K/AKT cascade was up-regulated by radiation, apatinib pretreatment effectively inhibited activation of PI3K/AKT pathway (Fig. 4b). Moreover, RAD51, which plays a major role in homologous recombination repair (HRR) during DSBs repair [29], was decreased in apatinib plus irradiation group as compared to irradiation alone. Consistently, γ-H2AX still remained at a higher level in combined treatment compared to irradiation alone (Fig. 2c).

**Fig. 5:**
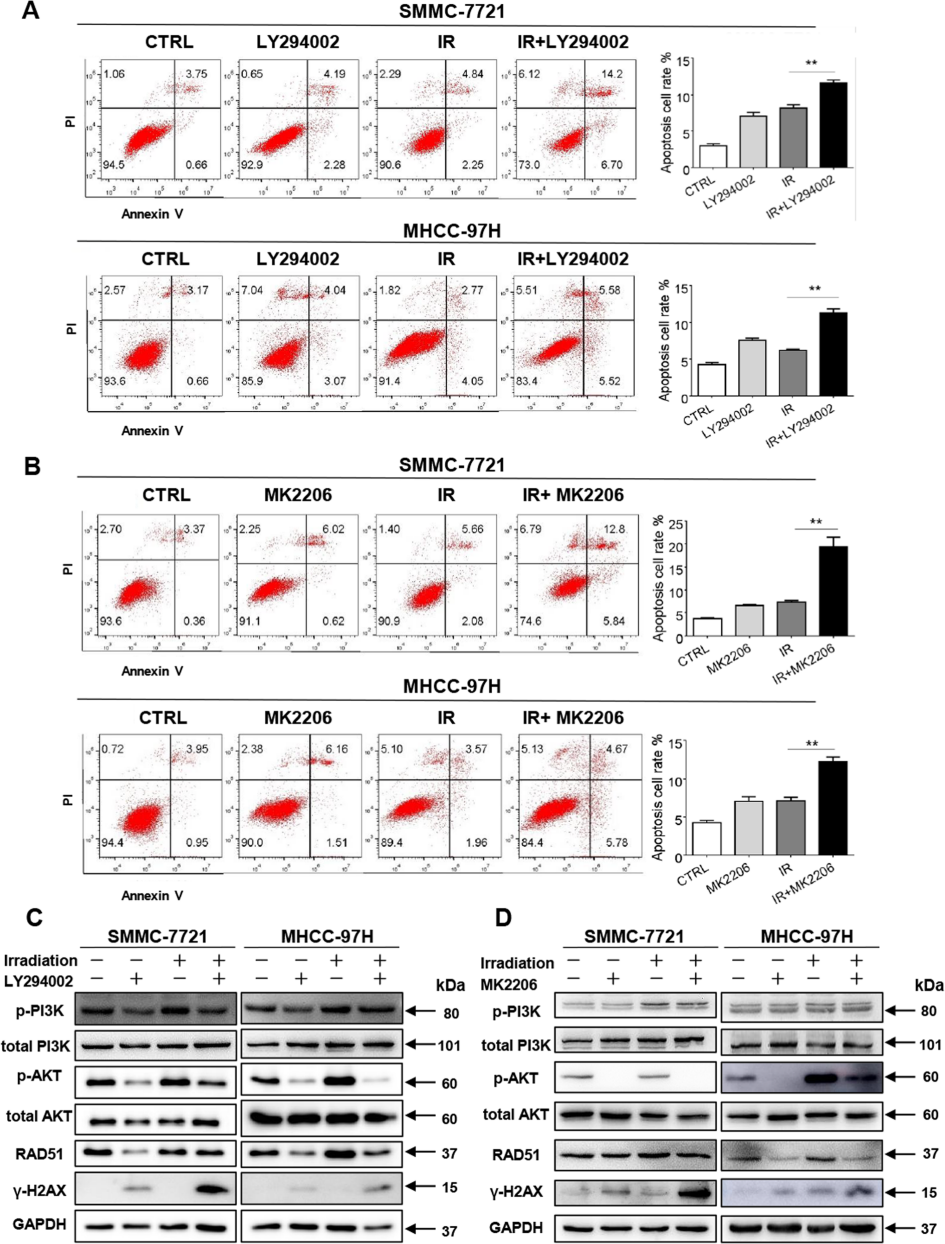
Inhibition of PI3K/AKT pathway contributes to the radiosensitization effect upon HCC cells. Under irradiation exposure, the effect of LY294002 (**a**) and MK2206 (**b**) on SMMC-7721 and MHCC97H cell apoptosis was assessed quantitatively by Annexin V-PI staining assays, respectively. **c**-**d** Protein expression of important genes in PI3K/AKT signaling and γ-H2AX were detected by Western blot at 24 h after IR

To further confirm whether the radiosensitization effect of apatinib was attributed to inhibition of PI3K/AKT pathway, LY294002, a highly selective inhibitor of PI3K, and MK2206, an allosteric inhibitor of AKT, were used for further study. The effect of PI3K or AKT inhibitors plus irradiation on apoptosis was assessed quantitatively by Annexin V-PI staining. Inhibition of PI3K plus irradiation markedly increased cell apoptosis as compared to irradiation alone in both SMMC-7721 and MHCC-97H  cell lines (Fig. 5a). Similarly, AKT inhibitor plus irradiation significantly enhanced cell apoptosis in both cell lines (Fig. 5b). Consistent with previous reports, irradiation upregulated the phosphorylated PI3K and phosphorylated AKT, while inhibition of PI3K diminished the irradiation-induced effects and decreased RAD51 level in both SMMC-7721 and MHCC-97H cell lines(Fig. 5c). Suppression of AKT with inhibitor showed similar effect of the phosphorylated AKT diminishment and RAD51 down-regulating (Fig. 5d). Taken together, these data indicated that apatinib sensitized HCC to radiation via suppression of PI3K/AKT pathway.

### Apatinib enhances xenograft tumor growth delay in mice receiving radiotherapy

**Fig. 6:**
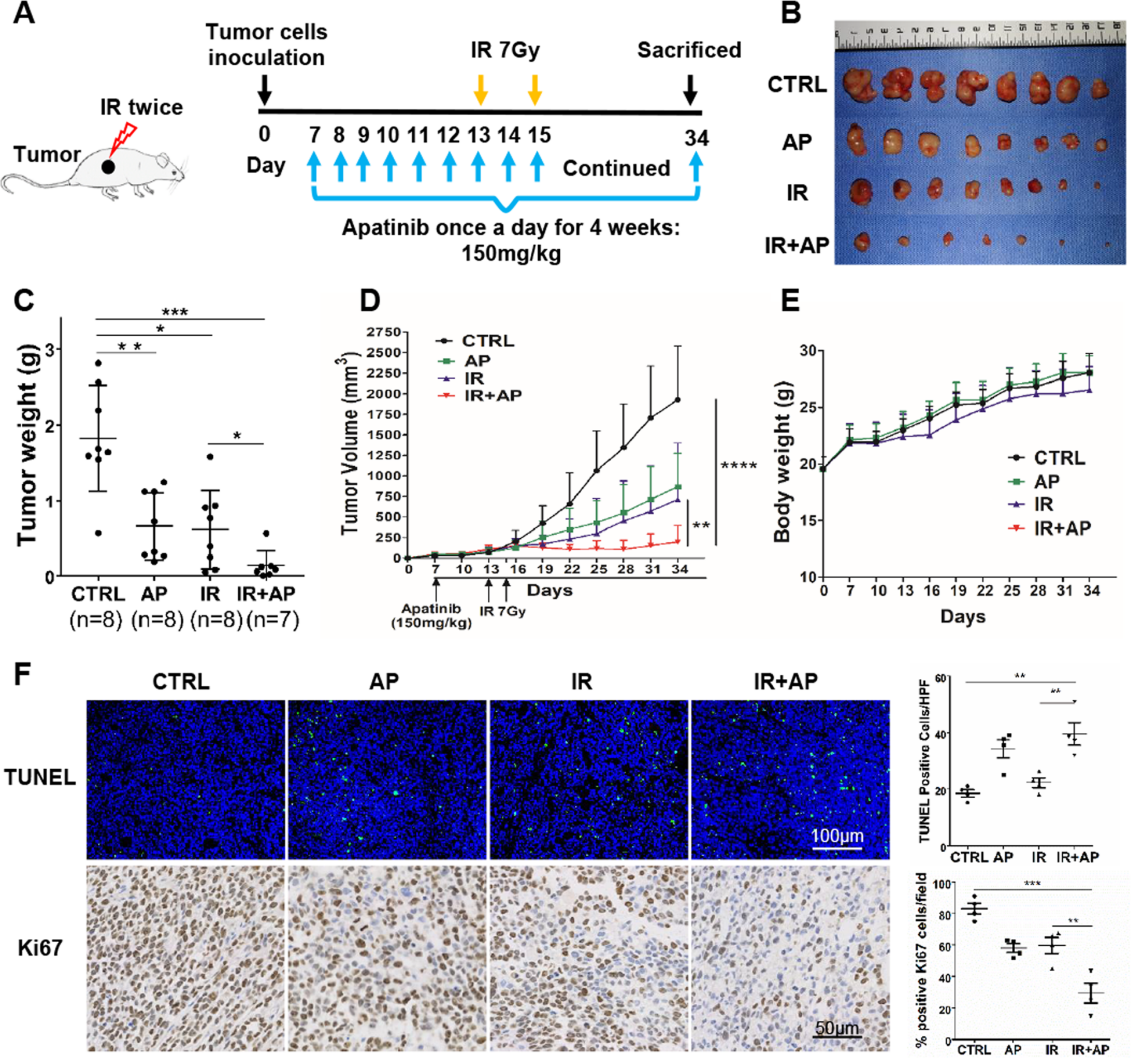
The radiosensitization effect of apatinib on HCC through in vivo xenograft tumor model. **a** The diagram of therapy design was showed. At day 0, SMMC7721 tumor cells were subcutaneously inoculated in the right flanks of mice. Mice were randomly divided into four groups when mice first developed a palpable mass ( 4mm in diameter) at day 7: Ctrl (*n* = 8), apatinib (*n* = 8), irradiation (*n* = 8), and irradiation with apatinib (*n* = 7). From day 7 onwards, apatinib was given gavage consecutively for 4 weeks with a daily dosage of 150 mg/kg until the end point at day 34. At day 13, irradiation (7 Gy) was delivered for the first time and the second one was conducted at day 15. Tumor xenografts (**b**), tumor weight (**c**), the growth curves (**d**) of each group were shown. **e** Body weight was measured during the treatment and shown as mean ± SEM. **f** The TUNEL and Ki67 staining showed that combination treatment group presented with significantly increased cell apoptosis and reduced expression of proliferation marker Ki67. *p < 0.05, ** *p* < 0.01, *** *p <* 0.001

SMMC-7721 xenograft models were established to further verify the radiosensitization effect of apatinib on HCC. SMMC-7721 cells were subcutaneously inoculated into the right flanks of BABL/c nude mice. Mice were randomly divided into four groups when mice first developed a palpable mass (4 mm in diameter): Ctrl (*n* = 8), apatinib (*n* = 8), irradiation (*n* = 8), and irradiation with apatinib (*n* = 7). The diagram of therapy design was showed (Fig. 6a). As expected, tumor weight and tumor volumes were mildly decreased in apatinib or irradiation group (0.6556 g ± 0.1586 or 0.616 g ± 0.1849) compared to 1.823 g ± 0.2472 in ctrl group (*p*<0.01, *p*<0.01, respectively). The irradiation combined with apatinib group effectively suppressed the growth of SMMC-7721 xenograft tumor models compared to irradiation treatment alone (at day 34) (apatinib plus irradiation vs. irradiation alone: 0.14 g ± 0.07221 vs. 0.616 g ± 0.1849, *p*<0.05, Fig. 6b-d). Besides, the combination treatment was well tolerated in all animals as it did not significantly affect body weight gain patterns in mice (Fig. 6e). Moreover, compared to radiotherapy group, combination treatment group presented with significantly increased TUNEL staining of cell apoptosis and significantly reduced expression of proliferation marker Ki67 (Fig. 6f). These results indicated that apatinib acted as a radiosensitizer in HCC in vivo, which was consistent with the in vitro data described above.

## Discussion

SBRT [5] and selective internal radiation therapy (SIRT) using yttrium-90 microspheres [30] have shown favorable therapeutic effects on HCC. In order to improve the efficacy and safety of radiotherapy, many radiosensitizers have been combined with radiotherapy [31]. Preclinical and clinical studies have shown that anti-angiogenic drugs, such as sorafenib and sunitinib [32], can increase the sensitivity of the radiotherapy effect on cancer. Apatinib, as a highly selective VEGFR2 inhibitor, has potential in the treatment of advanced HCC. However, there is no report to evaluate whether apatinib can enhance the therapeutic effect of radiotherapy on HCC. In our study, we found that radiotherapy combined with apatinib presented a synergistic anti-tumor effect through both in vitro and in vivo assays, which was mediated by induction of cell apoptosis and inhibition of PI3K/AKT pathway.

Previous studies have shown that the fraction size of 4–8 Gy and the total dose of 30–40 Gy are tolerable for patients with HCC, so 4 Gy, an effective and safe dose, was chosen for our in vitro study. We observed that the reproducing ability of HCC cells was dose-dependently inhibited by radiation as detected via colony formation assay (Fig. 1f-i). In combination with apatinib, the inhibition of reproduction ability caused by radiotherapy was more obvious, indicating that apatinib can sensitize HCC cells to radiotherapy (Fig. 1f-i). Increased apoptosis in cancer cells plays an important role in the efficacy of radiotherapy. In present study, apatinib combined with 4 Gy radiation significantly increased apoptosis as detected by Annexin V/PI and flow cytometry assay (Fig. 3a-d). Furthermore, the protein level of cleaved PARP, cleaved caspase-9 and cleaved caspase-3 was notably elevated in the combination group (Fig. 3e). Consistent with the in vitro results, apoptosis induced by radiation could be remarkably enhanced by the administration of apatinib in subcutaneous tumor model (Fig. 6f). Moreover, several studies reported that apatinib itself was capable of inducing tumor cell apoptosis, including anaplastic thyroid carcinoma [33], colorectal cancer [34], and osteosarcoma [25]. Therefore, our study demonstrated that apatinib may increase tumor radiation sensitivity by inducing cell apoptosis.

Radiation-related cell pro-survival signaling pathways include suppression of apoptosis induction, cell cycle arrest and promotion of DNA repair, all of which synergistically protect tumor cells from radiation damage ultimately [35]. Transcriptome sequencing was performed on two HCC cell lines treated with the combination therapy or the radiotherapy alone in present study. By using the pathway enrichment analysis, we identified PI3K/AKT signaling pathway was involved in both HCC cell lines (Fig. 4a). We observed that radiation could promote cancer cell survival through igniting the PI3K/AKT pathway, which, however, could be effectively inhibited by administration of apatinib. And, apatinib could downregulate RAD51 expression, which is a dysfunction marker of DNA repair (Fig. 4b). In fact, a considerable number of studies [36–38] have demonstrated that inhibition of PI3K/AKT signaling pathway could enhance the radiosensitivity of tumor cells both in vitro and in vivo. In addition, increased sensitivity to radiation by inhibiting PI3K/AKT pathway is associated with a decreased DNA repair capacity.

Although many radiosensitizers have been studied in HCC, the clinical applications so far have not been satisfactory [39]. As previous studies reported, antiangiogenic drugs sensitized tumors to radiotherapy through normalization of tumor blood vessels [32, 40]. Tumor vessels are characterized as high vessel density, tortuosity, dilation, chaotic, and lack of pericytes coverage, which cause tumor microenvironment hypoxia [41, 42]. Radiation depends on oxygen presence to generate free radicals to kill tumor cells and inadequate oxygen perfusion may attenuate radiotherapy effect [43]. Antiangiogenic drugs could normalize tumor blood vessels, reduce vessel density and improve oxygen perfusion so as to augment radiation effect. This normalization effect exerted most at about 1 week’s optimal time window after drug administration [42]. Given the above evidences, in the present study, we designed our treatment schedule to perform irradiation therapy at day 6 and day 8 after the first delivery of apatinib in mice xenograft tumor model. We observed that tumor blood vessel density decreased significantly in irradiation plus apatinib treatment group as compared to monotherapy group or control group (Additional file 3: Figure S2).

In HCC treatment, sunitinib has been showed with more adverse events even with several treatment-related deaths [44]. When compared with sorafenib, not only was sunitinib significantly inferior to sorafenib, but also sunitinib had more adverse events and toxicity [45]. However, as a first line drug for advanced HCC, sorafenib, when combined with radiotherapy, was reported to cause some fatal side effect to several patients [11]. What’s more, several studies illustrated that radiation sensitization effect of sorafenib should be attributed to inhibition of VEGFR-2 [46, 47]. Apatinib is a highly selective VEGFR-2 tyrosine kinase inhibitors, which was confirmed as a safe and effective concurrent treatment in HCC [15]. In this aspect, apatinib may have advantage over sorafenib and sunitinib as a radiosensitizer in HCC. Thus, in present study, we investigated apatinib as a radiosensitizer in HCC and found synergistic antitumor effect of apatinib and radiotherapy on HCC through inhibiting PI3K-AKT signaling pathway.

## Conclusions

In conclusion, the present study demonstrates that apatinib enhances the radiosensitivity of HCC through both in vitro and in vivo assays*,* and PI3K/AKT signaling pathway plays a critical role in mediating this effect. Our finding indicates the therapeutic potential of apatinib as a radiosensitizer for HCC.

## Supplementary information

**Additional file 1: Table S1.** Antibodies used for western blotting, immunohistochemistry and immunofluorescence.

**Additional file 2: Figure S1.** The effect of apatinib plus irradiation on cell cycle progression. SMMC-7721, MHCC-97H, HCCLM3 and Hep-3B cells were treated with or without apatinib for 24 h prior to exposure to 4 Gy irradiation. After 12 h, cells were collected for cell cycle analysis through flow cytometry. The radiation-induced G2/M-phase arrest was further enhanced by combination treatment in SMMC-7721 cell line, while such effect didn’t exist in other three cell lines.

**Additional file 3: Figure S2.** The effect of apatinib combined with radiotherapy on vascular density in mice xenograft tumor tissues. Representative fields and quantitative analysis of CD31 immunohistochemistry staining were showed. Vascular density determined by CD31 staining in mice tumor tissues was significantly decreased in combined strategy group as compared with monotherapy group or control group. *p < 0.05, ** *p* < 0.01, *** *p* < 0.001.

## Data Availability

All data generated or analysed during this study are included in this published article (and its supplementary information files).

## Abbreviations

CCK-8: Cell counting kit-8
CMC: Carboxymethyl cellulose
DMEM: Dulbecco’s modified Eagle’s media
DMSO: Dimethyl sulfoxide
DSBs: Double-strand breaks
FBS: Fetal bovine serum
HCC: Hepatocellular carcinoma
HRR: Homologous recombination repair
IHC: Immunohistochemistry
RFA: Radiofrequency ablation
RT: Radiotherapy
SBRT: Stereotactic body radiotherapy
SER: Sensitizing enhancement ratio
SIRT: Selective internal radiation therapy
TKI: Tyrosine kinase inhibitor
VEGFR2: Vascular endothelial growth factor receptor-2
γ-H2AX: Phosphorylated histone H2AX
