# Supplementary material for: Apatinib potentiates irradiation effect via suppressing PI3K/AKT signaling pathway in hepatocellular carcinoma
Source: J Exp Clin Cancer Res. 2019 Nov 6;38:454. doi: 10.1186/s13046-019-1419-1 (PMC6836669; doi:10.1186/s13046-019-1419-1)
Supplement: Supplementary file 1 — Additional file 1: Table S1. Antibodies used for western blotting, immunohistochemistry and immunofluorescence. [file 13046_2019_1419_MOESM1_ESM.docx]

| Antibodies used for Western blotting |  |  |  | |
| --- | --- | --- | --- | --- |
| Antibody | Dilution | Catalog # | | Vendor |
| Phospho-PI3 Kinase p85 (Tyr458)/p55 (Tyr199) | 1:1000 | 4228 | | Cell Signaling |
| PI3 Kinase p101 | 1:1000 | 5569 | | Cell Signaling |
| Phospho-Akt (Ser473) | 1:1000 | 4060 | | Cell Signaling |
| AKT | 1:1000 | 9272 | | Cell Signaling |
| Phospho-p44/42 MAPK(Thr202/Tyr204) | 1:1000 | 4370 | | Cell Signaling |
| p44/42 MAPK | 1:1000 | 9102 | | Cell Signaling |
| Caspase 3 | 1:1000 | 19677-1-AP | | Proteintech |
| Caspase 9/p35/p10 | 1:1000 | 10380-1-AP | | Proteintech |
| Cleaved PARP (Asp214) | 1:1000 | 5625 | | Cell Signaling |
| PARP | 1:1000 | 13371-1-AP | | Proteintech |
| Phospho-Histone H2A.X (Ser139) | 1:1000 | 9718 | | Cell Signaling |
| Rad51 | 1:1000 | ab133534 | | Abcam |
| GAPDH | 1:1000 | 2118 | | Cell Signaling |
| β-Tubulin | 1:1000 | 2128 | | Cell Signaling |
|  |  |  | |  |
|  |  |  | |  |
| Antibody used for immunohistochemistry |  |  | |  |
| Antibody | Dilution | Catalog # | | Vendor |
| Ki67 | 1:1000 | ab15580 | | Abcam |
|  |  |  | |  |
| Antibody used for immunofluorescence |  |  | |  |
| Antibody | Dilution | Catalog # | | Vendor |
| Phospho-Histone H2A.X (Ser139) | 1:500 | 9718 | | Cell Signaling |

**Table S1**
