# Supplementary material for: Apatinib potentiates irradiation effect via suppressing PI3K/AKT signaling pathway in hepatocellular carcinoma
Source: J Exp Clin Cancer Res. 2019 Nov 6;38:454. doi: 10.1186/s13046-019-1419-1 (PMC6836669; doi:10.1186/s13046-019-1419-1)
Supplement: Supplementary file 2 — Additional file 2: Figure S1. The effect of apatinib plus irradiation on cell cycle progression. SMMC-7721, MHCC-97H, HCCLM3 and Hep-3B cells were treated with or without apatinib for 24 h prior to exposure to 4 Gy irradiation. After 12 h, cells were collected for cell cycle analysis through flow cytometry. The radiation-induced G2/M-phase arrest was further enhanced by combination treatment in SMMC-7721 cell line, while such effect didn’t exist in other three cell lines. [file 13046_2019_1419_MOESM2_ESM.docx]

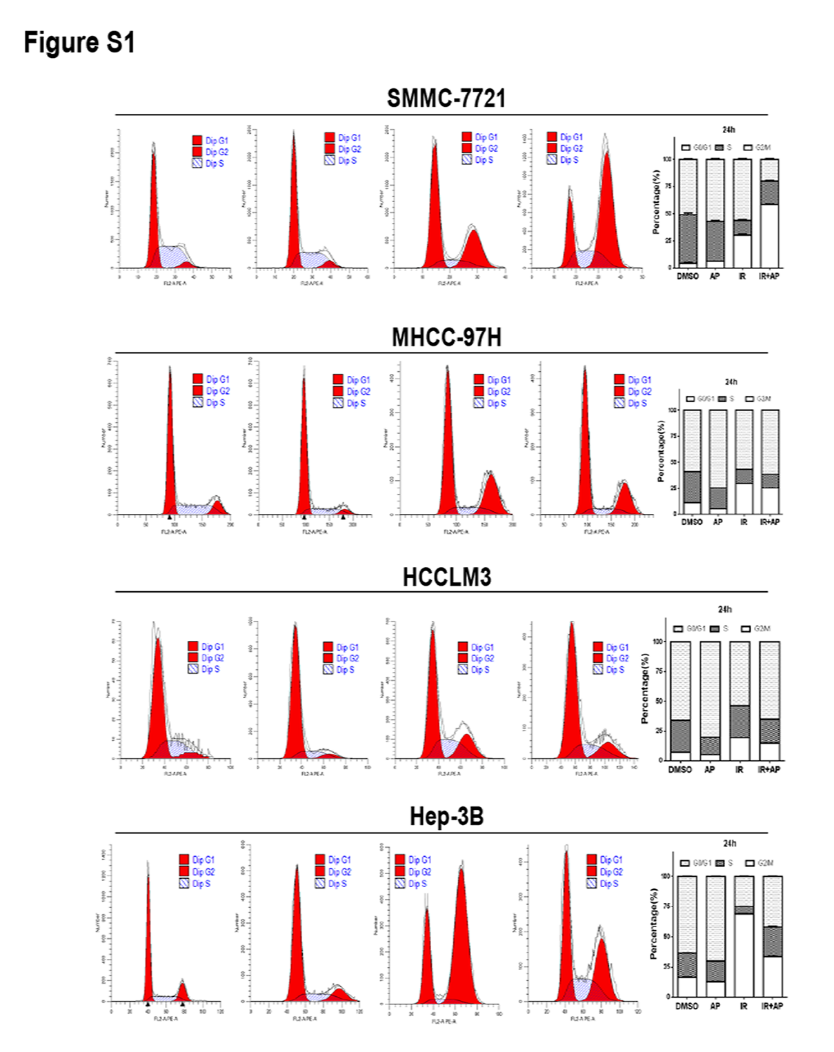


**Figure S1. The effect of apatinib plus irradiation on cell cycle progression**. SMMC-7721, MHCC-97H, HCCLM3 and Hep-3B cells were treated with or without apatinib for 24 h prior to exposure to 4 Gy irradiation. After 12 h, cells were collected for cell cycle analysis through flow cytometry. The radiation-induced G2/M-phase arrest was further enhanced by combination treatment in SMMC-7721 cell line, while such effect didn’t exist in other three cell lines.
