# Supplementary material for: Apatinib potentiates irradiation effect via suppressing PI3K/AKT signaling pathway in hepatocellular carcinoma
Source: J Exp Clin Cancer Res. 2019 Nov 6;38:454. doi: 10.1186/s13046-019-1419-1 (PMC6836669; doi:10.1186/s13046-019-1419-1)
Supplement: Supplementary file 3 — Additional file 3: Figure S2. The effect of apatinib combined with radiotherapy on vascular density in mice xenograft tumor tissues. Representative fields and quantitative analysis of CD31 immunohistochemistry staining were showed. Vascular density determined by CD31 staining in mice tumor tissues was significantly decreased in combined strategy group as compared with monotherapy group or control group. *p < 0.05, ** p < 0.01, *** p < 0.001. [file 13046_2019_1419_MOESM3_ESM.docx]

**Figure S2.** **The effect of apatinib combined with radiotherapy on vascular density in mice xenograft tumor tissues.** Representative fields and quantitative analysis of CD31 immunohistochemistry staining were showed. Vascular density determined by CD31 staining in mice tumor tissues was significantly decreased in combined strategy group as compared with monotherapy group or control group. **p*<0.05, ** *p*<0.01, *** *p*<0.001.
